# *KCNE1 *D85N polymorphism — a sex-specific modifier in type 1 long QT syndrome?

**DOI:** 10.1186/1471-2350-12-11

**Published:** 2011-01-18

**Authors:** Annukka M Lahtinen, Annukka Marjamaa, Heikki Swan, Kimmo Kontula

**Affiliations:** 1 Research Program for Molecular Medicine, Biomedicum Helsinki, University of Helsinki, Helsinki, Finland; 2 Department of Medicine, University of Helsinki, Helsinki, Finland; 3 Department of Cardiology, University of Helsinki, Helsinki, Finland

## Abstract

**Background:**

Long QT syndrome (LQTS) is an inherited ion channel disorder manifesting with prolongation of the cardiac repolarization phase and severe ventricular arrhythmias. The common *KCNE1 *D85N potassium channel variant prolongs QT interval by inhibiting I_Ks _(KCNQ1) and I_Kr _(KCNH2) currents and is therefore a suitable candidate for a modifier gene in LQTS.

**Methods:**

We studied the effect of D85N on age-, sex-, and heart rate-adjusted QT-interval duration by linear regression in LQTS patients carrying the Finnish founder mutations *KCNQ1 *G589D (n = 492), *KCNQ1 *IVS7-2A>G (n = 66), *KCNH2 *L552S (n = 73), and *KCNH2 *R176W (n = 88). We also investigated the association between D85N and clinical variables reflecting the severity of the disease.

**Results:**

D85N was associated with a QT prolongation by 26 ms (SE 8.6, *p *= 0.003) in males with *KCNQ1 *G589D (n = 213), but not in females with G589D (n = 279). In linear regression, the interaction between D85N genotype and sex was significant (*p *= 0.028). Within the *KCNQ1 *G589D mutation group, *KCNE1 *D85N carriers were more often probands of the family (*p *= 0.042) and were more likely to use beta blocker medication (*p *= 0.010) than non-carriers. The number of D85N carriers in other founder mutation groups was too small to assess its effects.

**Conclusions:**

We propose that *KCNE1 *D85N is a sex-specific QT-interval modifier in type 1 LQTS and may also associate with increased severity of disease. Our data warrant additional studies on the role of *KCNE1 *D85N in other genetically homogeneous groups of LQTS patients.

## Background

Long QT syndrome (LQTS) is an inherited arrhythmia disorder associated with risk of *torsades de pointes*, ventricular fibrillation, and sudden death. LQTS is caused by mutations of the ion channel genes controlling the repolarization phase of the cardiac action potential cycle [1,2]. The potassium channel KCNE1, also known as minK, regulates both the voltage-gated slowly activating I_Ks _potassium channel [3,4], encoded by the *KCNQ1 *gene, and the rapidly activating I_Kr _potassium channel [5], encoded by the *KCNH2 *gene. Mutations in *KCNE1 *underlie the LQT5 subtype of LQTS [6] and account for approximately 3% of known LQTS mutations [7]. In homozygous form, *KCNE1 *mutations may cause sensorineural hearing loss in association with LQTS, or Jervell-Lange-Nielsen syndrome [8].

A common variant D85N of *KCNE1 *was originally detected by Tesson *et al. *[9]. This variant has subsequently been shown to slow I_Ks _potassium channel, when studied in *Xenopus *oocytes [10], and to exhibit significant loss-of-function effects on both the KCNQ1- and KCNH2-mediated potassium currents, as measured in Chinese hamster ovarian cells [11]. In general population, *KCNE1 *D85N has a minor allele frequency of 0.8-1.4% [11-13] and is associated with a significant prolongation of the electrocardiographic QT interval [12-14]. Moreover, D85N has been detected in many LQTS patients as a second variant in addition to a more severe mutation [10,11], as well as in some individuals with drug-induced LQTS [15].

These findings prompted us to study whether *KCNE1 *D85N would also modify the QT interval and/or the clinical picture in patients with genetically homogeneous forms of LQTS in which the variability caused by the disease-causing mutation itself can be controlled for. To this end, we took advantage of the unique situation in Finland where four different mutations account for approximately 70% of the known spectrum of LQTS genes [16] and where the prevalence of molecularly defined LQTS appears to be the highest in the world [17]. Our results indicate a sex-specific QT-prolonging effect for D85N in *KCNQ1 *mutation carriers.

## Methods

### Patient cohort

The study sample consisted of all available (n = 712) carriers of the Finnish LQTS founder mutations, including 492 carriers of *KCNQ1 *G589D (5 of them also carried *KCNH2 *R176W, 1 *KCNH2 *L552S, and 1 a non-founder nonsense mutation *KCNQ1 *Y171X), 66 carriers of *KCNQ1 *IVS7-2A>G (1 of them also carried *KCNH2 *R176W), 73 carriers of *KCNH2 *L552S (2 of them homozygous), and 88 carriers of *KCNH2 *R176W. These four founder mutations account for 70%, and *KCNQ1 *G589D alone 50%, of all Finnish LQTS patients with an established molecular diagnosis of LQTS [16]. The collective prevalence of these four founder mutations in the general Finnish population is as high as 0.4% [17].

Subjects taking any known QT-prolonging medication at the moment of electrocardiogram (ECG) recording were excluded from the study. QT intervals were measured manually by a single cardiologist (H.S.) using the mean of two consecutive QT intervals in lead II in standard 12-lead ECG. Heart rate-corrected QT (QTc) was calculated using the Bazett's formula (QT/√RR). The study was performed in accordance with the Declaration of Helsinki and written informed consent was obtained from all participants. The Ethics Review Committee of the Department of Medicine, University of Helsinki, approved the study.

### Molecular genetic analyses

*KCNE1 *253G>A (D85N, rs1805128) was genotyped in DNA from venous blood samples using polymerase chain reaction with primers 5'-GAGATTGGAGTGGTGGATGGA-3' and 5'-CACCCCTTACAACAGCCAAAA-3' followed by Lwe I digestion (Fermentas, Ontario, Canada) and agarose gel electrophoresis. Previously identified heterozygous and major and minor homozygous samples served as controls in the assay.

### Statistical analyses

Normality of continuous distributions was reviewed visually. Non-normally distributed age was normalized by Blom's method. The effect of *KCNE1 *D85N on age-, sex-, and heart rate (RR interval) -adjusted QT interval was studied by linear regression analysis using 1-df additive model (genotype coded as D85D = 0, D85N = 1, N85N = 2). Because gender differences were detected, an additional model included also a multiplicative interaction term between *KCNE1 *D85N genotype and sex. In *KCNQ1 *G589D carriers, association of D85N with proband/family member status (information available for all 492 G589D carriers), appearance of syncope (information available, n = 488), use of beta blocker medication (information available, n = 347), and occurrence of pacemaker or implantable cardioverter-defibrillator (information available, n = 349) was studied by Fisher exact test. Statistical analyses were performed with SPSS 17.0 (Statistical Package for Social Sciences, SPSS Inc., Chicago, IL, USA). A *p *value < 0.05 was considered statistically significant.

## Results

### Effect of *KCNE1 *D85N on QT interval in LQTS founder mutation carriers

Of the 712 LQTS founder mutation carriers, 689 had the *KCNE1 *genotype D85D, 21 had the heterozygous genotype D85N, and 2 were minor homozygotes (N85N) (Table 1). The frequency of the N85 allele was 1.8%, which is slightly higher than the corresponding frequency in the general Finnish population (1.4%) [12] and in other reported populations (0.8-1.0%) [11,13]. Of the 21 D85N heterozygotes, 15 had the *KCNQ1 *G589D mutation, 2 had *KCNH2 *L552S, 3 had *KCNH2 *R176W, and 1 was a double heterozygote for the *KCNQ1 *G589D and *KCNH2 *R176W mutations. None of the *KCNQ1 *IVS7-2A>G mutation carriers had the minor N85 allele. The two N85N minor homozygotes (boys aged 4 and 10) were *KCNQ1 *G589D carriers and were siblings from the same family. As a whole, the founder mutation carriers were derived from 126 LQTS families. The number of *KCNE1 *D85N carriers in type 2 LQTS was too small for statistical comparisons and therefore the analyses were confined to *KCNQ1 *G589D carriers and the combined group of founder mutation carriers.

**Table 1:** The clinical characteristics of the study sample

	D85D major homozygotes	D85N heterozygotes	N85N minor homozygotes
All subjects			
n (%)	689 (96.8)	21 (2.9)	2 (0.3)
Age (years)	29.7 ± 20.0	28.7 ± 20.2	7.0 ± 4.2
Males (%)	280 (40.6)	8 (38.1)	2 (100)
QTc (ms)	458 ± 36.8	472 ± 35.2	468 ± 34.6
Founder mutation status			
*KCNQ1 *G589D			
n	474^a^	16^b^	2
QTc	459 ± 36.2	479 ± 31.0	468 ± 34.6
*KCNQ1 *IVS7-2A>G			
n	66^b^	0	0
QTc	465 ± 30.0	-	-
*KCNH2 *L552S			
n	71^c^	2	0
QTc	464 ± 48.3	490 ± 7.1	-
*KCNH2 *R176W			
n	84^d^	4^e^	0
QTc	445 ± 30.6	442 ± 46.5	-

The figures represent the mean ± SD. SD, standard deviation; QTc, heart rate-corrected QT interval.

^a^Four subjects carrying also *KCNH2 *R176W, one *KCNH2 *L552S, and one *KCNQ1 *Y171X.

^b^One subject carrying also *KCNH2 *R176W.

^c^Includes two *KCNH2 *L552S homozygotes and one subject carrying also *KCNQ1 *G589D.

^d^Four subjects carrying also *KCNQ1 *G589D and one *KCNQ1 *IVS7-2A>G.

^e^One subject carrying also *KCNQ1 *G589D.

**Figure 1:**
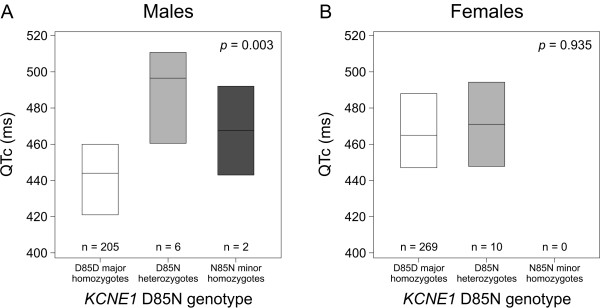
**QTc-interval duration in different *KCNE1 *D85N genotype classes in males and females with *KCNQ1 *G589D**. Box plots show medians and interquartile ranges. The *p *values in linear regression analyses using additive genotypic model are shown for (A) males and (B) females separately. For interaction between D85N genotype and sex, *p *= 0.028. QTc, heart rate-corrected QT-interval.

Patients with *KCNQ1 *G589D (n = 492) was the largest group of founder mutation carriers. A QT-prolonging effect of 26 ms (SE 8.6, *p *= 0.003) was detected in males with G589D (n = 213), whereas D85N did not prolong QT interval in females with G589D (n = 279) (Table 2, Figure 1). In our cohort, all male *KCNQ1 *G589D carriers with *KCNE1 *N85 (n = 8) were of age ≤16. To account for the possible confounding effect of age, male G589D carriers ≤16 years were analyzed also as a separate group. Even in this analysis, the effect of N85 allele on QT interval remained essentially the same (+23 ms, SE 8.8, *p *= 0.010). The majority (9 out of 10) of the female *KCNQ1 *G589D carriers with *KCNE1 *D85N were adults ≥18 years (median age 34 years, range 0-59 years). In order to further study the gender specificity of D85N, a multiplicative interaction term for D85N genotype and sex was introduced into the linear model. This interaction term received a significance of *p *= 0.028, indicating that D85N indeed has a sex-specific effect for QT interval in *KCNQ1 *G589D carriers.

**Table 2:** Effect of *KCNE1 *D85N on age-, sex-, and heart rate-adjusted QT interval

	Total n	n with D85N	Effect (ms)	SE (ms)	*p *value
All founder mutation carriers	712	23	+13.1	6.0	0.028
Males^a^	290	10	+20.1	7.7	0.010
Females^a^	422	13	+1.6	9.1	0.857
*KCNQ1 *G589D carriers	492	18	+16.9	6.3	0.007
Males^a^	213	8	+25.7	8.6	0.003
Females^a^	279	10	+0.7	9.2	0.935

SE, standard error.

^a^QT interval adjusted by age and heart rate.

When all founder mutation carriers were considered together, *KCNE1 *D85N was associated with a 13 ms (SE 6.0) prolongation of QT interval per each N85 minor allele (*p *= 0.028, Table 2). A similar gender-specific analysis revealed that this association was confined to males (Table 2).

### Association of *KCNE1 *D85N with selected clinical variables

**Figure 2:**
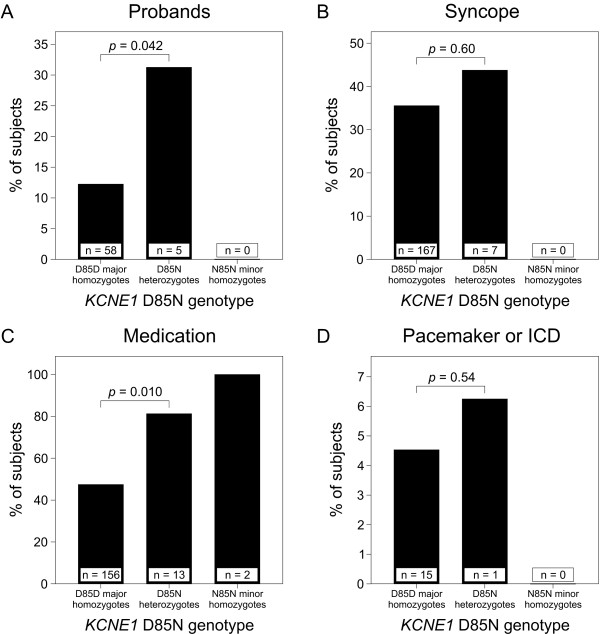
**Association of the *KCNE1 *D85N variant with selected clinical variables in *KCNQ1 *G589D carriers**. Percentage of (A) probands, (B) patients having experienced syncope, (C) patients with beta blocker medication, and (D) patients with pacemaker or ICD, in different *KCNE1 *D85N genotype classes. The *p *value between D85N heterozygotes and D85D major homozygotes in Fisher exact test is shown above the columns and the number of cases in each genotype group inside the columns. ICD, implantable cardioverter-defibrillator.

The clinical importance of the LQTS-modifying effect of *KCNE1 *D85N was studied in the largest founder mutation group of *KCNQ1 *G589D carriers. In these subjects, the proband/family member status, occurrence of syncope, use of beta blocker medication, and use of pacemaker or implantable cardioverter-defibrillator were compared between the D85N heterozygotes and non-carriers (Figure 2). Of the 16 D85N heterozygotes, 5 (31%) were probands, compared to 58 (12%) of the 474 D85D homozygotes (*p *= 0.042). The proportion of subjects using beta blocker medication was also higher in D85N heterozygotes (81%) than in D85D homozygotes (47%, *p *= 0.010). The median age at which the medication was started was 15 years (range 0-58 years) for the D85N heterozygotes and 8 years (range 5-11 years) for the N85N minor homozygotes. A similar but non-significant trend for difference between non-carriers and heterozygotes was observed in the occurrence of syncope and use of pacemaker or implantable-cardioverter defibrillator (Figure 2).

## Discussion

The *KCNE1 *D85N variant has been shown to lead to a substantial QT-interval prolongation in the general population [12-14]. In fact, we are not aware of any other gene variant showing a population-based frequency of >1% and an equally large effect on QT interval. In order to test the effect of this variant on cardiac repolarization in patients with LQTS, we studied the association of *KCNE1 *D85N to QT interval in a homogeneous group of Finnish LQTS founder mutation carriers. We found that the minor N85 allele significantly prolongs age-, sex-, and heart rate-adjusted QT interval in type 1 LQTS patients with the mutation G589D. An interaction between D85N genotype and sex was detected (*p *= 0.028), and a sex-specific analysis revealed that the N85 allele prolongs QT interval by 26 ms in males but not in females (Table 2). Our material is too small to draw conclusions of the role of D85N in LQTS type 2 caused by *KCNH2 *mutations.

In this study, we detected a gender difference in the effect of *KCNE1 *D85N on QT interval in type 1 LQTS. We reported previously that the occurrence of the N85 minor allele was associated with a 10 ms prolongation of the QT interval in the general Finnish population [12]. We carried out a retrospect analysis of our population sample [12] and found that the effect of D85N on QT interval was slightly larger in males (+11.3 ms) than in females (+10.0 ms). Gender is known to influence QT interval at the population level, with females in general showing longer QT intervals than males [18], and there are sex- and age-specific differences in risk of arrhythmias in the different subtypes of LQTS [19,20]. It is of note that also *KCNQ1 *G589D presents a gender difference since the female carriers have >20 ms longer QTc compared to the male carriers [21], a finding replicated in the present study (Figure 1, D85D homozygotes). *KCNE1 *D85N appears to invert this sex difference. Interestingly, Friedlander *et al. *[22] found that G38S, another polymorphism of *KCNE1*, was associated with QT-interval variation in healthy males but not in females.

Sex differences in cardiac repolarization appear to result from both transcriptional and non-transcriptional regulatory mechanisms. In female mice hearts, *KCNE1 *mRNA is more abundant compared to males [23]. Differences in KCNE1 protein levels may therefore influence the sex-specific sensitivity to *KCNE1 *variants. Sex hormones have also been reported to alter the gating kinetics of cardiac ion channels and to modulate the effects of channel blocking agents [24,25]. For example, 17β-oestradiol inhibits I_Ks _[24] and I_Kr _[25] channels and enhances the effect of a KCNH2 blocker [25]. It is therefore possible that *KCNE1 *D85N interferes with the binding of sex-specific I_Ks _channel regulators, but the exact mechanisms underlying the sex difference reported in the present study remain to be explored.

In previous studies, *KCNE1 *D85N has been found to associate with congenital LQTS [10,11]. Compound carriers of two or more LQTS mutations have a longer heart rate-corrected QT interval (QTc) and a more severe phenotype than carriers of only one mutation [10,26]. *KCNE1 *D85N has also been proposed to cause LQTS in the absence of any documented mutation in known LQTS genes, but the age at onset may be higher and QTc shorter than in patients with a more severe LQTS mutation [11]. *In vitro *experiments indicate that *KCNE1 *N85 significantly reduces both I_Ks _and I_Kr _currents, thus delaying repolarization in mammalian cells [11]. This gene variant also contributes to loss of I_Ks _function together with a *KCNQ1 *mutation in *Xenopus *oocytes [10]. In another study of mammalian cells, however, D85N did not affect I_Ks _current when co-expressed with KCNQ1 [27].

In the present study, we found that the QT-prolonging effect of *KCNE1 *D85N is substantially larger in males with the LQTS mutation *KCNQ1 *G589D (26 ms) than in the general population (10 ms). This result suggests that *KCNQ1 *and *KCNE1 *mutations and variants may interact with each other, at least in males, to produce an even more pronounced QT-prolonging effect than when occurring separately. This interaction could be caused by the coassembly of the two proteins to form a functional I_Ks _channel. However, due to small number of subjects with the *KCNE1 *N85 allele and LQT1 mutations other than *KCNQ1 *G589D, we cannot draw conclusions on the general applicability of this effect in LQT1, nor provide sufficient data on a possible similar relation in LQT2. It should be emphasized that the association between D85N and QT interval in type 1 LQTS should be replicated in another material. However, we realize that this is not an easy task considering the population prevalence of D85N. Thus, any replication material should contain hundreds of genetically uniform patients with type 1 LQTS.

Our study also provides evidence that the *KCNE1 *D85N variant may be associated with a more severe phenotype of type 1 LQTS, as the proportions of probands and users of beta blocker medication were significantly higher in D85N heterozygotes than in non-carriers (Figure 2). Indirectly, these associations suggest that D85N carriers may seek medical help more often than non-carriers. A similar but non-significant trend was detected in the occurrence of syncope and use of pacemaker or implantable cardioverter-defibrillator. Paulussen *et al. *[15] identified 2 carriers of the N85 allele among 32 patients with drug-induced LQTS, while no such carriers were observed in the series of 34 patients with *torsades de pointes *studied by Mank-Seymour *et al. *[28]. Sotoodehnia *et al. *[29] suggested that D85N could be associated with a higher death rate in males but not in females. Clearly, more extensive population-based studies are required in which the role *KCNE1 *D85N variation as determinant of life-threatening and fatal arrhythmias will be explored. Polymorphisms in another gene, *NOS1AP*, have also been associated with QT prolongation and cardiac events in LQTS patients [30].

## Conclusions

Our study suggests that *KCNE1 *D85N variation has a gender-dependent QT-prolonging effect in *KCNQ1 *G589D mutation carriers and could thus complicate the symptoms of LQTS. Previously, this variant has not been studied *in vivo *in a large cohort of LQTS mutation carriers. As *KCNE1 *D85N is relatively frequent occurring in 2-3% of the general population, this interaction between *KCNQ1 *and *KCNE1 *could ultimately have direct implications in counseling of LQTS patients. However, future studies are required to assess the direct risks of arrhythmias and/or sudden death in LQTS patients carrying *KCNE1 *N85.

## Competing interests

The authors declare that they have no competing interests.

## Authors' contributions

AML participated in the design of the study, performed the molecular genetic and statistical analyses, and drafted the manuscript. AM participated in the design of the study, data analysis, and writing of the manuscript. HS collected the patient material and participated in the design of the study, data analysis, and writing of the manuscript. KK participated in the design of the study, data analysis, and writing of the manuscript. All authors read and approved the final manuscript.

## Pre-publication history

The pre-publication history for this paper can be accessed here:

http://www.biomedcentral.com/1471-2350/12/11/prepub
